# Detection of a major Lynch Syndrome-causing *MLH1* founder variant in a large-scale genotyped cohort

**DOI:** 10.1007/s10689-024-00400-4

**Published:** 2024-06-07

**Authors:** Lauri J. Sipilä, Mervi Aavikko, Janne Ravantti, Samantha Martin, Teijo Kuopio, Laura Lahtinen, FinnGen, Päivi Peltomäki, Jukka-Pekka Mecklin, Lauri A. Aaltonen, Toni T. Seppälä

**Affiliations:** 1 Department of Medical and Clinical Genetics, University of Helsinki, Biomedicum Helsinki Haartmaninkatu 8), PO Box 63, 00014 Helsinki, Finland; 2 Applied Tumor Genomics, Research Programs Unit, University of Helsinki, Biomedicum Helsinki Haartmaninkatu 8), PO Box 63, 00014 Helsinki, Finland; 3 Finnish Cancer Registry, Unioninkatu 22, 00130 Helsinki, Finland; 4 Institute for Molecular Medicine Finland (FIMM), HiLIFE, University of Helsinki, Helsinki, Finland; 5 Molecular and Integrative Biosciences Research Programme, Faculty of Biological and Environmental Sciences, University of Helsinki, FI‐00014 Helsinki, Finland; 6 Central Finland Biobank, Wellbeing Services County of Central Finland and University of Jyväskylä, Jyväskylä, Finland; 7 Department of Pathology, Wellbeing Services County of Central Finland, Jyväskylä, Finland; 8 HUSLAB Laboratory of Genetics, HUS Diagnostic Center, HUS, Helsinki University Hospital, 00029 Helsinki, Finland; 9 Faculty of Sport and Health Sciences, University of Jyväskylä, Jyväskylä, Finland; 10 Department of Surgery, Nova Hospital, Jyväskylä, Finland; 11 iCAN Digital Precision Cancer Medicine Flagship, University of Helsinki, 00290 Helsinki, Finland; 12 Faculty of Medicine and Health Technology, Tampere University, Tampere, Finland; 13 Tays Cancer Centre, Tampere University Hospital, Tampere, Finland; 14 Department of Gastrointestinal Surgery, Helsinki University Central Hospital, University of Helsinki, Helsinki, Finland; 15 Department of Gastroenterology and Alimentary Tract Surgery, Tampere University Hospital, Tampere, Finland

**Keywords:** Lynch syndrome, Mismatch repair deficiency, Biobank, Genotype association, Personalized medicine

## Abstract

Some 50% of Finnish Lynch Syndrome (LS) cases are caused by a founder variant in *MLH1*, in which the entire exon 16 has been lost due to an Alu-mediated recombination event. We piloted detecting the variant in FinnGen, a large genotyped cohort comprising approximately 10% of the current Finnish population, and validated the *MLH1* founder variant status of identified individuals residing in the Central Finland Biobank catchment area. A consensus sequence flanking the deletion was identified in whole genome sequences of six LS individuals with the founder variant. Genotype data of 212,196 individuals was queried for regional matches to the consensus sequence. Enrichment of cancer and age at cancer onset was compared between matching and non-matching individuals. Variant status was validated for a subset of the identified individuals using a polymerase chain reaction assay. Allelic matches in a chosen target region was detected in 348 individuals, with 89 having a cancer diagnosis (Bonferroni-adjusted p-value = 1), 20 a familial cancer history (p-adj. < .001), with mean age of onset of cancer being 53.6 years (p-adj. = .002). Eighteen of potential variant carriers had been sampled by the Central Finland Biobank, of which four (22%) were validated as true variant carriers. The workflow we have employed identifies *MLH1* exon 16 deletion variant carriers from population-wide SNP genotyping data. An alternative design will be sought to limit false positive findings. Large genotyped cohorts provide a potential resource for identification and prevention of hereditary cancer.

## Introduction

Lynch Syndrome (LS) is an autosomal dominant cancer syndrome, caused by pathogenic variants in genes involved in DNA mismatch repair: *MLH1*, *MSH2*, *MSH6*, and *PMS2*. LS confers a significant increase in an individual’s cancer risk, with lifetime risk reaching some 80% in carriers of pathogenic variants of *MLH1* and *MSH2 *[1]. LS diagnosis enables targeted screening for cancers contributing to the LS spectrum, providing an opportunity for early tumor diagnosis and intervention, while chemotherapeutic preventative strategies can be applied as well [2]. Approximately half of all known Finnish LS cases are caused by a founder variant in *MLH1*, characterized by a genomic deletion of exon 16 that has emerged through an Alu-mediated recombination event [3].

FinnGen is a research project which produces versatile data types from biobanked samples and connects these with clinical information from national registries, aiming to cover 500,000 Finns by the end of the project [4]. The core of the project’s biological data consists of raw DNA microarray data and imputed genotypes. The genetic data consists of both legacy data from older studies and new data produced specifically for the project. The project provides a means to identify clinically relevant genetic information on a national scale.

When individuals donate a sample to a biobank in Finland, they sign a consent form and specify their will on whether they can be contacted if clinically relevant information is discovered from their sample. To provide such information in the context of LS we are preparing to initiate RETURN, a study which aims to identify individuals harboring pathogenic LS variants in FinnGen for subsequent genotype validation. Individuals with a positive result will be then contacted for recruitment into the next phase of the study, in which their familial cancer history and their response to receiving life-altering information from biobank data will be surveyed. The individuals will also be instructed to contact the Finnish LS registry for enrollment, which supports LS individuals in accessing special health care.

While efforts were made to design probes to capture the Finnish population-specific *MLH1* exon 16 deletion variant through the FinnGen DNA microarray, the task proved difficult due to the lack of specificity in the region following the fusion of two Alu sites. Despite being the most significant cause of LS in Finland, the variant’s allele frequency in the population is too low for conventional imputation methods in FinnGen data with currently available resources. We designed an alternative approach for identifying potential *MLH1* exon 16 deletion variant carriers in FinnGen, based on detecting associated alleles flanking the deletion by utilizing whole genome sequence (WGS) data from known LS individuals with the variant as a reference. The performance of the approach was investigated in matching individuals residing in the catchment area of Central Finland Central Hospital.

### Methods

**Fig. 1:**
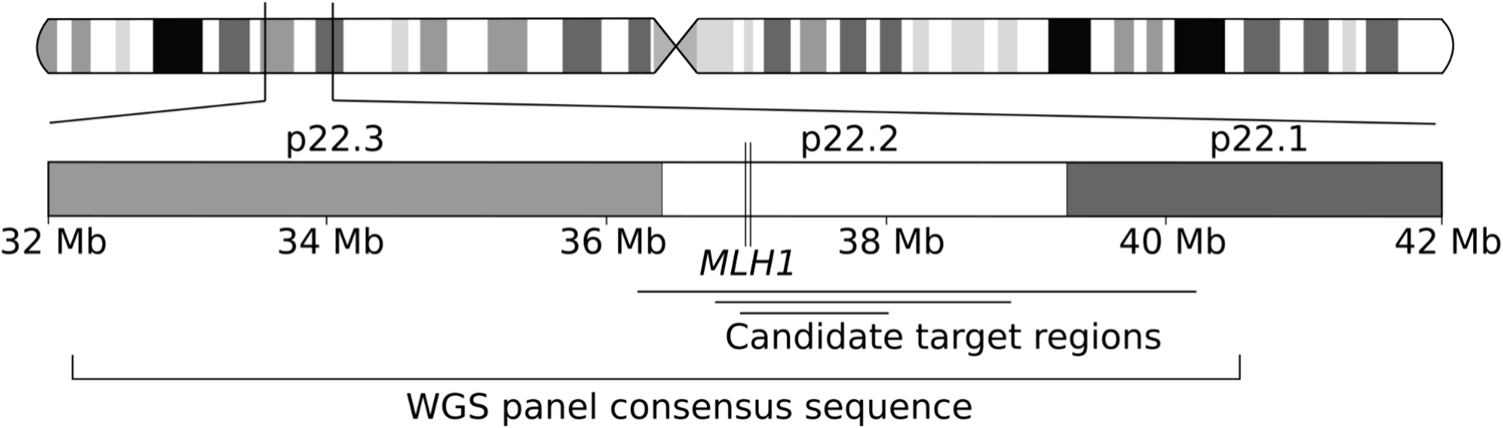
The positions of the *MLH1* gene, WGS panel consensus sequence, and three alternative candidate target regions in chromosome 3 for the identification of potential variant carriers. Each candidate target region overlaps the entire *MLH1* gene (chr3:36,993,226–37,050,896), including the deletion site at exon 16 (chr3:37,044,577–37,048,113). The deletion site is not emphasized in the figure due to its small size relative to the gene and the complete region of interest. Mb = megabase

Using GATK’s HaplotypeCaller v. 4.1.9, we produced base pair-resolution variant call format files from normal tissue WGS data of six known *MLH1* exon 16 deletion carriers for the GRCh38 reference sequence chromosome 3 region 27,000,000–47,000,000. In this panel, we identified a consensus sequence flanking the deletion, starting from the deletion site at chromosome 3 37,044,577–37,048,113. Loci with a genotype quality score less than 50 in any sample were ignored. This consensus sequence, spanning chromosome 3 32,193,122–40,483,917 (Fig. 1), was assumed to be representative of the founder haplotype sequence.

Potential carrier identification and statistical analysis was carried out using FinnGen’s Data Freeze 8 (N = 342,499 individuals), filtering out samples from National Institute of Health legacy studies due to difficulties in re-contacting the individuals for the potential return of genetic information. Furthermore, legacy data has been produced with different genotyping platforms than the rest of the FinnGen samples. Thus, the final number of individuals in the analysis was 212,196. The FinnGen microarray design had probes for 224 biallelic single nucleotide variants (SNV) in the chromosome 3 region 32,302,363–40,269,834. Exploratory analyses indicated only a limited association of cancer endpoints with the p-arm telomeric end of the founder haplotype. Thus, we narrowed the region of interest to chr3:36,347,797–40,269,834 through which an increase in cancer endpoint enrichment was observed. We further segmented this region into 2402 partially overlapping candidate target allele sets based on the available SNV probes in the FinnGen array for the region. Each candidate allele set encompassed all consecutive alleles corresponding to the founder haplotype in that subregion, with all such sets containing all alleles within ± 100,000 base pairs from the deletion. For each allele set, we identified individuals in FinnGen with an identical match of heterozygous or homozygous genotypes. Two-sided Fisher’s exact tests were calculated to determine the association of allele set positivity with history of familial cancer (ICD-10 code Z80; N = 3228) and the number of first primary cancer diagnoses (N = 70,448). Association with the age of onset of the first cancer was calculated with a two-sided Mann–Whitney U test. Allele set-negative individuals with information for the tested variable were used as controls in each analysis. Bonferroni correction was applied to account for multiple testing. Identification and association analyses were carried out in the FinnGen Sandbox using BCFtools v. 1.18, and Python packages SciPy v. 1.2.3, statsmodels v. 0.11.0 and pandas v. 0.24.2.

Personal identifiers of the individuals matching the final selected target region were subsequently cross-referenced with the Finnish LS registry to identify any previously known LS carriers. The founder variant statuses of samples from matching individuals available in the Central Finland Biobank were determined with a PCR assay modified from Nyström-Lahti et al., 1995. The PCR reaction includes two forward primers, one specific for the mutant allele (5ʹ-GAGCCTCCAATACAATGTTGAATAGAAG-3ʹ) and another specific for the wild-type allele (5ʹ-ACATATGTGACATCCTCTCCACTCC-3ʹ), plus a common reverse primer (5ʹ-CTCTCCATGTCTGGGTCCTTC-3ʹ). An amplification product of 271 base pairs identifies the mutant allele and a 112 base pair-fragment the wild-type allele.

After validating the carrier status of these individuals, the length of the sequence match to the WGS panel consensus sequence flanking the deletion site was determined for each sample. These were compared between deletion carriers and wild type (WT) individuals.

## Results

Cancer-related endpoints were enriched in individuals sharing sets of consecutive allele matches with the WGS panel consensus sequence flanking the deletion, and three of the alternative allele sets were chosen for further consideration (Table 1). Familial cancer history was statistically significantly associated with all such allele sets, while the cancer diagnosis age remained statistically significant after correction for multiple testing in allele sets covering shorter regions, which had larger numbers of matching individuals. The number of individuals with cancer did not remain statistically significant after correcting for multiple testing.

**Table 1:** Cancer endpoint associations of individuals matching the WGS consensus sequence at different genomic regions

Region size (Mb)	From	To	Allele set size	No. of matches	Cancer (any)			Familial cancer history			Cancer diagnosis age	
					N	OR	p (adj.)	N	OR	p (adj.)	mean	p (adj.)
7.97^a^	32,302,363	40,269,834	224	11	5	3.36	1	6	117.8	< .001	49.50	1
3.92	36,347,797	40,269,834	119	19	10	4.48	1	10	122.99	< .001	48.58	1
2.05	36,852,557	38,906,537	61	73	23	1.86	1	16	30.36	< .001	45.36	.011
1.69^b^	36,937,156	38,630,069	46	348	89	1.39	1	20	7.03	< .001	53.57	.002

A subset of a total of 2403 allele sets is presented. P-values adjusted with the Bonferroni method. *Mb* megabase, *N* number of individuals in the study cohort matching each allele of the consensus sequence in the region, *OR*, odds ratio

^a^Statistics for the longest possible target region of 7.97 Mb included for reference, as it was not included in the considered allele sets due to a small number of matches﻿

^b^Region chosen for deletion carrier validation pilot

The shortest of the alternative allele sets was chosen for the validation pilot. Of the 348 individuals identified as matching this allele set in FinnGen Data Freeze 8, 19 had provided a sample to FinnGen via the Central Finland Biobank. Cross-referencing of the personal identifiers of these individuals with the Finnish LS registry by the Central Finland Biobank revealed that four of them were already present in the registry (21% of the Central Finland Biobank matches). Eighteen of these 19 individuals had an available DNA sample at the biobank and these were used to validate the putative *MLH1* founder variant status by PCR, yielding a positive result for the same previously known four carriers (22% of samples), while the rest were negative.

Studying the maximum consecutive allele match lengths flanking the deletion site in a sample-specific manner revealed that all deletion carriers matched the WGS consensus sequence starting from at least chromosome 3 position 35,155,278, with each of them having an allelic match at the end of the complete target region at position 40,269,834 (mean matching region length of 7,254,242 base pairs). An allele set encompassing all variants in this target region would identify 13 possible carriers in the complete data set. WT individuals often matched the WGS consensus in the p-arm telomeric end, while the last allelic match to the WGS consensus in the whole group occurred at locus 39,105,233 (mean matching region length of 4,776,482 base pairs).

## Discussion

This pilot investigation serves as a proof-of-concept for producing potentially life-saving information for the subsequent return to biobank participants, through secondary use of large-scale genetic research data. The *MLH1* exon 16 deletion founder variant was chosen as the target for this pilot due to it being the most significant causative LS variant in Finland, while being virtually undetectable with standard DNA microarray analysis. Our method approximated conventional genotype imputation methods, with the advantage that it did not require us to include the WGS of known affected individuals in the haplotype phasing reference panel or re-run resource-intensive imputation calculations. This approach can be applied for the detection of similar variants in genotyped cohorts if sufficient reference data from affected individuals is also available.

The enrichment of true positives among the potential carrier group demonstrates that our employed method is working as intended, and showcases the translational potential of large-scale genotyped cohorts in general. Comparing the consensus sequence match lengths of the true and false positives may allow us to improve the method for future efforts. To this effect, we have designed a new allele set extending further towards the centromere which only captures a subset of variants with preferred minor allele frequency in the range of 1–5%, as these are more informative of the haplotype than more common variants. Removing uninformative common alleles from the allele set may remove some false negatives, as the requirement of uninterrupted consecutive matches will lead to a negative result even from a single false variant call.

We were unable to calculate the sensitivity and specificity of the assay, as we did not possess information on the true mutation carrier status produced using a gold standard method for the complete cohort. For example, there may remain variant carriers in the FinnGen cohort who were not detected as positive with this method. Similarly, we only know the rate of true positive results for a selected regional subsample of the cohort.

Identification of only previously known carriers in this pilot is likely explained by the fact that the investigation used data from the Central Finland region, where cascade testing to identify LS individuals has been particularly thorough, and consequently the Finnish LS registry has a high regional coverage. In the upcoming RETURN study the identification effort will be expanded to other biobanks, and we expect to identify previously unknown carriers by analyzing samples from regions where LS has not been a research focus. The scope of the study extends beyond the *MLH1* exon 16 deletion founder variant, with another Finnish founder variant in the splice acceptor site of *MLH1* exon 6 being the next high-impact target. Rare pathogenic variants such as those listed in the InSiGHT database [5] may also be studied in large-scale genetic cohorts. However, the error rates present in microarray technologies, combined with the extremely low allele frequency of many of these variants, make genotype calls unreliable [6]. In such cases, determining the founder haplotype for the variant will likely improve identification efforts.

In conclusion, we have utilized genetic and clinical data in the FinnGen project framework to identify potential carriers of the high-impact *MLH1* exon 16 deletion variant. We conducted a validation pilot in potential carriers in Central Finland, where we identified 19 individuals. Four out of 18 tested were true positives and previously known to the Finnish LS registry. Implemented in the whole available population, the approach should facilitate the identification and management of LS variant carriers, and lead to earlier diagnosis of LS-related cancers. Similar approaches should also be feasible elsewhere, where large genotyped cohorts are available.

The FinnGen study has been evaluated by the Coordinating Ethics Committee of the Hospital District of Helsinki and Uusimaa (statement number HUS/990/2017).

The FinnGen study has been approved by Finnish Institute for Health and Welfare (permit numbers: THL/2031/6.02.00/2017, THL/1101/5.05.00/2017, THL/341/6.02.00/2018, THL/2222/6.02.00/2018, THL/283/6.02.00/2019, THL/1721/5.05.00/2019 and THL/1524/5.05.00/2020), Digital and population data service agency (permit numbers: VRK43431/2017–3, VRK/6909/2018–3, VRK/4415/2019–3), the Social Insurance Institution (permit numbers: KELA 58/522/2017, KELA 131/522/2018, KELA 70/522/2019, KELA 98/522/2019, KELA 134/522/2019, KELA 138/522/2019, KELA 2/522/2020, KELA 16/522/2020), Findata permit numbers THL/2364/14.02/2020, THL/4055/14.06.00/2020, THL/3433/14.06.00/2020, THL/4432/14.06/2020, THL/5189/14.06/2020, THL/5894/14.06.00/2020, THL/6619/14.06.00/2020, THL/209/14.06.00/2021, THL/688/14.06.00/2021, THL/1284/14.06.00/2021, THL/1965/14.06.00/2021, THL/5546/14.02.00/2020, THL/2658/14.06.00/2021, THL/4235/14.06.00/2021 and Statistics Finland (permit numbers: TK-53-1041-17 and TK/143/07.03.00/2020 (earlier TK-53-90-20) TK/1735/07.03.00/2021).

The Biobank Access Decisions for FinnGen samples and data utilized in FinnGen Data Freeze 8 include: THL Biobank BB2017_55, BB2017_111, BB2018_19, BB_2018_34, BB_2018_67, BB2018_71, BB2019_7, BB2019_8, BB2019_26, BB2020_1, Finnish Red Cross Blood Service Biobank 7.12.2017, Helsinki Biobank HUS/359/2017, Auria Biobank AB17-5154 and amendment #1 (August 17 2020), AB20-5926 and amendment #1 (April 23 2020), Biobank Borealis of Northern Finland_2017_1013, Biobank of Eastern Finland 1186/2018 and amendment 22 § /2020, Finnish Clinical Biobank Tampere MH0004 and amendments (21.02.2020 & 06.10.2020), Central Finland Biobank 1-2017, and Terveystalo Biobank STB 2018001.
